# Adaptive dietary and exercise strategies for weight loss in Adults with Prediabetes Trial (ADAPT): a sequential multiple assignment randomized trial

**DOI:** 10.1016/j.ajcnut.2025.07.034

**Published:** 2025-08-07

**Authors:** Katie M Ellison, Aseel El Zein, Navneet Kaur Baidwan, Christine C Ferguson, Lauren Adele Fowler, David R Bryan, Chelsi Reynolds, Dalton Hermanson, Kelly J Berg, Tapan Mehta, James O Hill, Holly R Wyatt, R Drew Sayer

**Affiliations:** 1 Department of Nutrition Sciences, University of Alabama at Birmingham, Birmingham, AL, United States; 2 Department of Family and Community Medicine, University of Alabama at Birmingham, Birmingham, AL, United States; 3 School of Medicine, University of Missouri - Kansas City, Kansas City, MO United States

**Keywords:** obesity, prediabetes, high-carbohydrate diets, reduced carbohydrate diets, time restricted eating, exercise, counseling, adaptive intervention, sequential multiple assignment randomized trial

## Abstract

**Background:**

Dietary carbohydrate restriction, time-restricted eating (TRE), and exercise are common strategies for weight loss and improving glycemic control. However, the optimal combination and sequence of these strategies is unclear.

**Objectives:**

We investigated adaptive treatment strategies for weight loss and improving cardiometabolic health in adults with overweight or obesity (BMI [in kg/m^2^] ≥27) and prediabetes.

**Methods:**

ADAPT was a 16-wk group-based weight loss Sequential Multiple Assignment Randomized Trial. In total, 83 adults were initially randomized to either a calorie-restricted reduced carbohydrate (RC) diet or high-carbohydrate (HC) diet. Nonresponders (<2.5% weight loss at week 4) were rerandomly assigned to augment initial dietary prescriptions with either TRE or exercise counseling.

**Results:**

Of 82 participants (53.8 ± 11.7 y; 84.3% females; BMI: 38.3 ± 7.2) who completed week 4 assessments, 46 (55.4%) were nonresponders and rerandomly assigned to TRE (*n* = 22) or exercise (*n* = 24). Weight loss at week 16 was similar between HC and RC (0.15 kg; 95% CI: −1.86, 2.16 kg; *P* = 0.88). Although the HC group showed greater improvements in fasting glucose, (−8.0 mg/dL; 95% CI: −15.29, −0.67 mg/dL), changes in A1c, fasting insulin, and quantitative insulin sensitivity check index were not different between HC and RC. Among nonresponders, assignment to second-stage interventions of TRE or exercise did not differentially affect changes in any study outcomes, and less weight loss was achieved among early nonresponders compared with responders despite the addition of TRE or exercise.

**Conclusions:**

The group-based program resulted in clinically significant weight loss that was similar between calorie-restricted HC and RC diets. However, counseling to follow a HC diet reduced fasting glucose compared with RC. Patients with obesity and prediabetes who are unable to achieve early weight loss may require more intensive and costly intervention strategies (i.e., meal provisions and supervised exercise) to improve obesity treatment outcomes.

This trial was registered at ClinicalTrials.gov as NCT04745572 (https://clinicaltrials.gov/study/NCT04745572?term=NCT04745572&rank=1).

## Introduction

Behavioral obesity interventions typically comprise the simultaneous delivery of multiple intervention components such as specific diet and exercise recommendations as well as a multitude of behavior change techniques to promote the adoption and maintenance of health behaviors. Despite their comprehensive nature, behavioral interventions are ineffective for many people and are characterized by considerable variability in achieved weight loss [1,2]. Further, the simultaneous delivery of several behavioral intervention components precludes the identification of the individual contributions of each component to the overall effect of the intervention package, and traditional randomized clinical trials cannot efficiently determine if, when, and under what conditions the intervention strategies should be modified based on early in-treatment outcomes. Conversely, the Sequential Multiple Assignment Randomized Trial (SMART) experimental approach was designed for developing and evaluating dynamic treatment regimens and enables the evaluation of individual treatment components [3]. This approach may enhance the translation and eventual implementability of behavioral interventions by identifying individuals who are likely to succeed with fewer and/or less costly intervention components and reserving more intensive and/or costly intervention components (e.g., supervised exercise and meal provisions) for individuals who do not meet treatment goals with initial strategies.

The lifestyle modification program used in the Diabetes Prevention Program (DPP) trial is representative of a highly effective but complex behavioral intervention package. The DPP used several strategies to promote an energy-restricted, low-fat diet and engaging in ≥150 weekly minutes of physical activity including 16 individually delivered behavioral counseling sessions over 24 wk, supervised exercise, individualized maintenance phase strategies, and a “toolbox” of intervention incentive strategies [4,5].The dietary advice in the DPP was characterized by low fat intake (25% of energy), moderate protein intake (15%–20% of energy) and relatively high-carbohydrate intake (55%–60% of energy). However, some evidence suggests that reducing carbohydrate intake is more effective than a low-fat, high-carbohydrate dietary pattern for weight loss, particularly in people with prediabetes or diabetes [6–11]. More recently, interventions targeting the within-day timing of food intake rather than the content of the dietary pattern have been investigated for weight loss and improving metabolic health. Time-restricted eating (TRE) involves constricting the time when food is consumed to a shortened window of typically 4–10 h/d and has been shown to produce modest weight loss and improved glycemic control, including in people with prediabetes [12–14].

Thus, a lifestyle intervention package based on the DPP and that also includes reduced carbohydrate (RC) intake and TRE could be an effective strategy for weight loss and improving cardiometabolic health for people with obesity and prediabetes. However, it is currently unclear which combination or sequence of these strategies is most effective. This study used the SMART experimental approach and a 16-wk group-based behavioral intervention to investigate adaptive treatment strategies comprising initial randomization to either follow a RC (~35% total energy from carbohydrate) or higher carbohydrate (HC, ~50% total energy from carbohydrate) dietary plan with a second randomization of early nonresponders to either receive 1-on-1 counseling to initiate a TRE program or to increase exercise participation. We hypothesized that greater weight loss (primary outcome), improved glycemic status [glucose, insulin, hemoglobin A1c (A1c), and quantitative insulin-sensitivity check index (QUICKI), secondary outcomes), and cardiometabolic health status (body fat, systolic blood pressure (SBP), diastolic blood pressure (DBP), total cholesterol (TC), HDL-cholesterol , LDL-cholesterol, and triglycerides, and tertiary outcomes] would be observed when people were initially assigned to RC diet compared with an HC diet and when nonresponders received individual exercise counseling compared with TRE counseling. We further hypothesized that an adaptive treatment regimen initiating with an RC diet and augmenting with exercise for nonresponders would produce the greatest weight loss and improvement in glycemic status.

## Methods

### Participants

**FIGURE 1.**
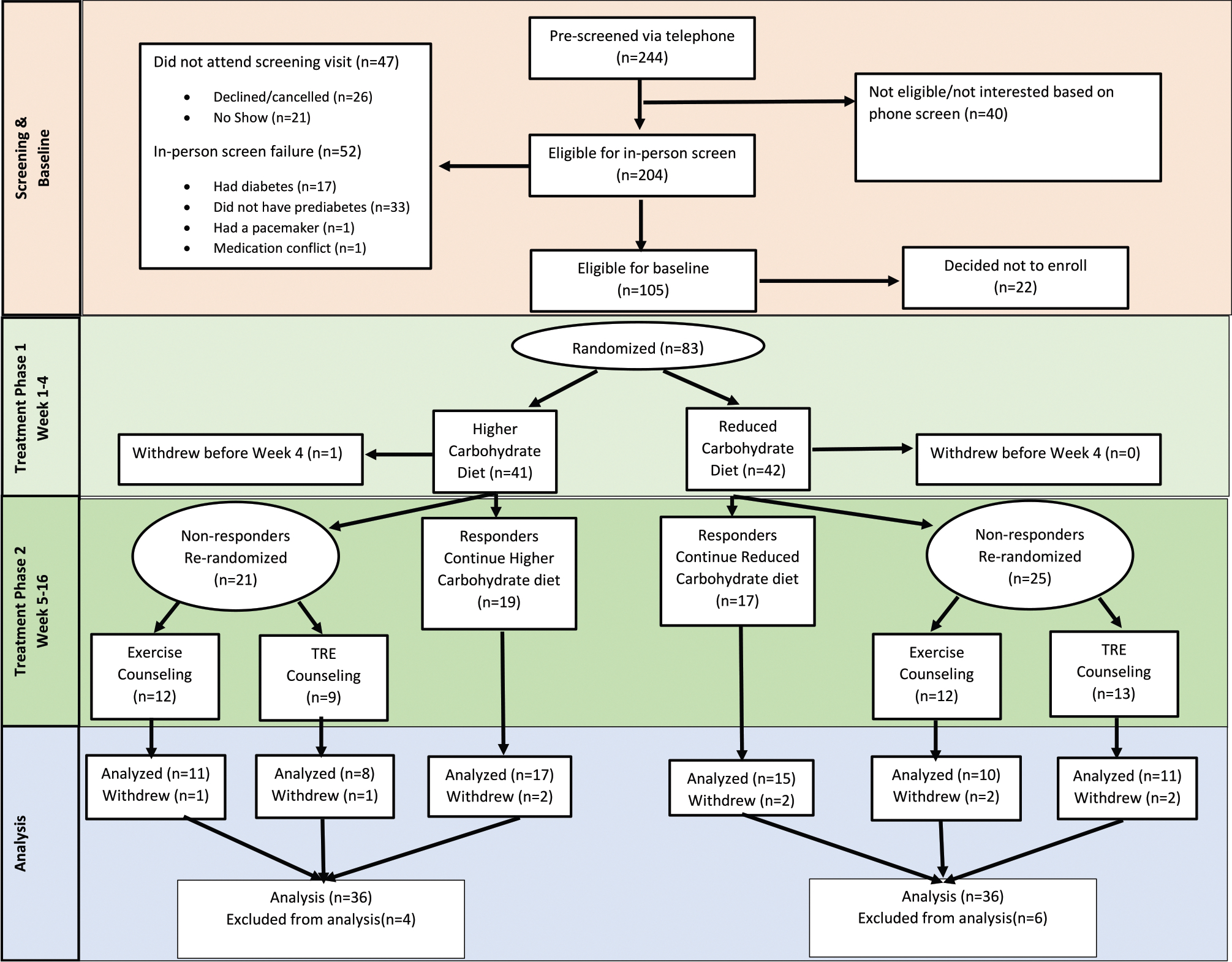
Study flow chart CONSORT diagram. CONSORT, Consolidated Standards of Reporting Trials, TRE, time-restricted eating.

Recruitment began in February 2022 and ended with the last follow-up visit in November 2023. Participants were eligible for inclusion if they were *1*) between the ages of 18–75 y old, *2*) had a BMI of ≥27, *3*) had prediabetes (fasting glucose of ≥100 mg/dL and/or A1c ≥5.7<6.5%), and *4*) had stable use of (or not taking) medications known to affect body weight. Participants were excluded if they were *1*) currently pregnant, previously pregnant within the past 6 mo, or planning to become pregnant within the next 6 mo, *2*) began taking weight loss medications or changed dosages within the last 3 mo, *3*) had a battery-operated implant such as a pacemaker, *4*) self-reported having an eating disorder or illicit substance use, or *5*) was taking exogenous insulin. Figure 1 illustrates the recruitment flow according to the CONSORT diagram [15]. This study was approved by the University of Alabama at Birmingham (UAB) Biomedical institutional review board, and all participants provided written informed consent to participate. The trial was registered at clinicaltrials.gov (NCT04745572). During the review of this manuscript, it came to the authors’ attention that the primary, secondary, and tertiary outcomes were not appropriately distinguished in the initial study entry on clinicaltrials.gov. The entry was corrected during the review of this manuscript in April 2025 to reflect weight change as the primary outcome upon which the study was statistically powered, changes in glycemic status as secondary outcomes due to the prediabetes inclusion criteria, and other cardiometabolic outcomes as tertiary.

### Study design

#### Randomization and sample size justification

Randomizations for the first-stage (HC or RC) and second-stage (exercise or TRE) interventions were stratified by sex and used permuted block randomization by sex with a minimum block size of 2 and a maximum block size of 8 by a statistician via REDCap. Participants were informed of their initial first-stage assignment at the baseline study visit. Research staff were not blinded to diet allocation. Participants were identified as responders/nonresponders at week 4, with nonresponders informed of their second-stage assignment within 1 wk of the week 4 assessment.

This SMART was powered based on the primary aim, which was to compare weight loss from baseline to week 16 between the HC and RC diets (i.e., main effect of first-stage intervention). Statistical power calculations were based on estimates from a previous group–based weight loss trial conducted by our research group [16]. In that trial, pooled weight loss at week 16 was 7.8% ± 5.7% and a difference of 3% weight loss between first-stage intervention strategies (HC compared with RC) was considered clinically meaningful [17]. Assuming a similar coefficient of variation in a hypothetical comparator group with a mean weight loss of 4.8% at week 16 and calculated SD of 3.5%, this resulted in a detectable effect size of Cohen *d* of 0.63, or medium effect for weight loss. It was determined that a sample size of 82 would be required to detect an effect of this size or larger at 80% power with a 2-tailed test at α = 0.05. The recruitment goal for this study was 90 participants, which would have provided *>*80% power to detect a difference of ≥3% weight loss. The anticipated nonresponse rate to the first-stage intervention was 67%, and based on unpublished data from a previous group-based weight loss trial [16]. Under that scenario, nonresponders were anticipated, and a priori power calculations indicated the SMART would provide *>*80% power to detect a medium-large effect size (*d* = 0.73) at α = 0.05 for the difference in weight loss between exercise and TRE (i.e., the main effect of second-stage interventions).

Power calculations and statistical analyses did not account for potential intervention-induced clustering that may result from an individually randomized group trial study design. However, the study was designed to reduce the potential effects of using a shared interventionist (agent) across multiple participants in the group-based weight loss classes, which included only participants assigned to either the HC or RC condition. Specifically, both weight loss classes within each cohort were led by a single interventionist, which resulted in a fully crossed, single-agent individually randomized group trial design that was expected to equally distribute the agent effect across both conditions with each cohort. Previous simulations studies have shown that intervention-induced clustering has a nominal effect on the type 1 error rate with this design [18]. In our own assessment of class-level weight loss (Supplemental Figure 1), no clear evidence of a class effect was observed for within-cohort, between-class weight loss data. However, a new interventionist was hired for the last cohort of the study (classes 5 and 6), and box plots indicate potentially greater weight loss in that cohort than that in cohorts 1 and 2. No difference in weight loss between classes 5 and 6 were observed.

#### Weight management program

All participants (*n* = 83) received a behavioral weight loss intervention with 16 weekly group-based classes held via Zoom lasting ~1 h each. Topics discussed each week involved behavior change strategies that were adapted from the Centers for Disease Control and Prevention PreventT2 curriculum, which is the current version of the DPP [19]. Topics for the group-based sessions included information on a wide array of topics, mostly focused on strategies for staying motivated and adhering to the diet plans (Supplemental Table 1). Weight loss classes were exclusive to participants of the same initial diet assignment, but the content of the classes was identical across conditions and were led by the same interventionist within each cohort as described earlier. Participants also received diet-specific counseling via 1-on-1 sessions with trained nutrition/lifestyle behavior interventionists.

#### Description of first-stage interventions

A cohort-based recruiting strategy was implemented with 3 cohorts of ~30 participants per cohort to comprise 2 classes with ~15 participants per class (1 HC and 1 RC). Study staff calculated participants’ energy needs using the Mifflin St. Jeor equation with the appropriate activity factor (AF) based on participants’ self-reported physical activity level (AF = 1.0–1.39, sedentary; AF = 1.4–1.59, low active; AF = 1.6–1.89, active; AF = 1.9–2.5, very active) [20]. Study staff follow-up with participants to assign them a trained research-based interventionist to serve as their main contact for study-related questions, scheduling visits, and providing participants with 5 sample daily meal plans based on their energy needs for weight loss (500- to 750-kcal restriction) consistent with their intervention assignment. Total energy from carbohydrates was ~35% in the RC diet with ~20 g of fiber and 50% in the HC diet with ~30 g of fiber. Both diet plans were low-fat (≤30% energy from fat) with an emphasis on unsaturated fats (i.e., nuts, seeds, and olive oil) and included foods generally aligned with current Dietary Guidelines for Americans (e.g., fruits, vegetables, whole-grains, low-fat dairy, and lean sources of protein). Total energy from protein was ~35% in the RC diet and 20% in the HC diet. The diabetic exchange list was also provided to use as a guide for making appropriate substitutions and their assigned individual nutrition interventionist explained how to use it [21].

**FIGURE 2.**
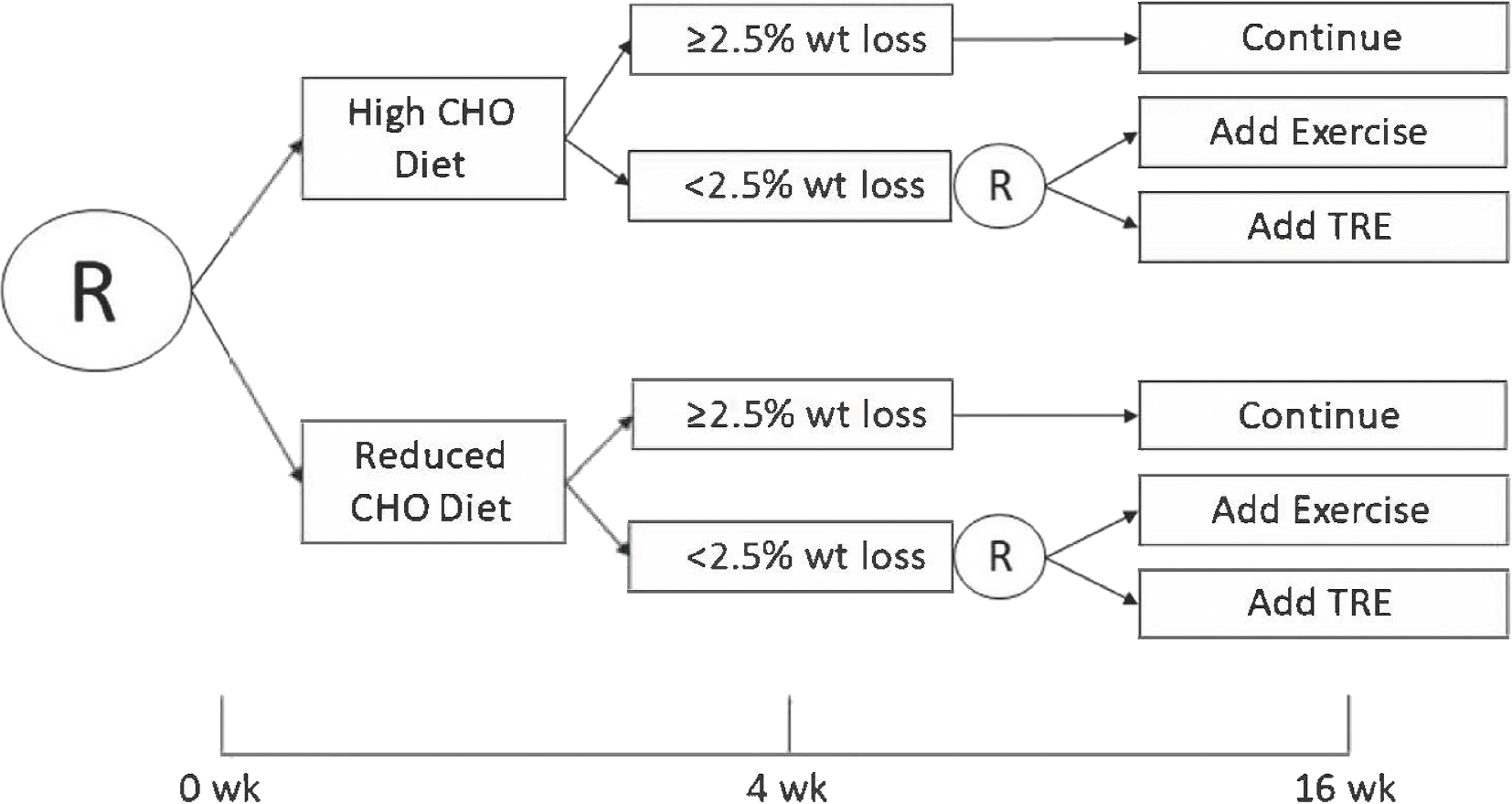
Sequential multiple assignment randomized trial schematic. CHO, carbohydrate; TRE, time-restricted eating.

Responders were defined as having achieved ≥2.5% weight loss at week 4 of the intervention and continued to receive their diet-specific counseling for the remaining 12 wk of the study. Participants with <2.5% weight loss at week 4 were identified as nonresponders and were rerandomly assigned to second-stage interventions of augmenting their diet plan with either 1-on-1 counseling to increase exercise or initiate TRE. Weight loss of 2.5% after 4 wk was also used in a previous weight loss SMART [22] and is likely representative of a true change in body weight rather than within the limits of random within-day variability [17]. A schematic of the SMART is shown in Figure 2.

#### Description of second-stage interventions

##### Time-restricted eating.

Nonresponding participants assigned to TRE were counseled to follow an early TRE protocol while continuing with their RC or HC dietary pattern. Participants were counseled to consume 4 meals during an 8-h window that began no later than 09:00. Outside of this period, the participant was instructed not to consume any calories but water and no-calorie beverages were allowed. Interventionists also discussed barriers (e.g., feelings of hunger, meeting dietary needs, and adherence during social situations), strategies for success, and motivations.

##### Exercise counseling.

Nonresponders assigned to receive exercise counseling were instructed to progressively increase moderate-to-vigorous physical activity to ≥150 min/wk. Participants were encouraged to achieve the weekly exercise goal through aerobic exercise, especially walking. In cases where the participant was unable or unwilling to walk (i.e., poor neighborhood accessibility and no gym access), they could choose from alternative activities such as resistance training, yoga, Pilates, and flexibility training to meet the 150-min goal. Participants met individually once weekly with an exercise specialist on Zoom outside of the weekly weight loss classes. Initial sessions with the exercise specialist were ~30 min and subsequent sessions were ~15–20 min. Time was spent discussing barriers, enablers, motivations, strategies for exercise, and goal setting.

### Study assessments and procedures

Study visits were conducted at the Fitness, Lifestyle, and Optimal Wellness Clinic housed within the UAB Family and Community Medicine Clinic or the UAB Wellness, Health, and Research Facility on the Lakeshore Foundation Campus. Participants were given intervention materials including a binder with weekly curricula that would be discussed in weight loss classes, a food scale, and a body weight scale that they were encouraged to use throughout the study. Participants arrived at baseline and week 16 study visits after an overnight fast. Body weight was measured using the Detecto-1000 or Eat Smart Precision Plus digital scales accurate to ±0.1 kg. At week 4, participants either met with their assigned interventionists on Zoom or took a picture of their weight using the scale they received at baseline to determine responder and nonresponder status. Blood pressure was measured using a clinical grade electronic sphygmomanometer (Welch Allyn 71WT-B Connex Spot). Fasting state blood samples were obtained from an antecubital vein and analyzed by the UAB Outreach Laboratory for glucose, A1c, insulin, and lipid panel at baseline and week 16. QUICKI was calculated from fasting glucose and insulin [23]. Glucose in serum plasma was analyzed using the Beckman Coulter AU System using the hexokinase G-6-PDH method [24]. The AU system has a linear range between 10 and 800 mg/dL for serum/plasma determinations. Samples exceeding the upper limit were diluted and repeated. During operation of the Beckman Coulter AU analyzer, ≥2 levels of an appropriate quality control material are tested a minimum of once a day. Additionally, controls are performed after calibration, with each new lot of reagents, and after specific maintenance or troubleshooting steps described in the AU User’s Guide. A1c was in serum plasma was analyzed using the Premier HgbA1c Analyzer, which has an analytical measurement range (AMR) between 3.8% and 18.5%. For results outside the AMR, estimated mean glucose is reported instead. It should be noted that all A1c’s in the present research were within the AMR. The Trinity Biotech Glycated Hemoglobin Controls (level 1 and level 2) are used to ensure quality control.

### Statistical analyses

All study data were collected and managed using REDCap electronic data capture tools hosted at UAB [25,26]. REDCap is a secure, web-based software platform designed to support data capture for research studies, providing *1*) an intuitive interface for validated data capture; *2*) audit trails for tracking data manipulation and export procedures; *3*) automated export procedures for seamless data downloads to common statistical packages; and *4*) procedures for data integration and interoperability with external sources. Statistical analyses were performed using SAS version 9.4 (SAS Institute) or R version 4.4.2 [27] (R Core Team, 2024). Change in body weight at week 16 was the primary outcome, changes in glycemic status (glucose, A1c, insulin, and QUICKI) were considered as secondary outcomes due to participants having prediabetes, and other cardiometabolic outcomes (body fat, blood pressure, and lipid panel) were considered as tertiary. All comparisons presented in the results are evaluated as change scores from baseline to week 16 time points, and all statistical tests were 2-tailed, with significance set at *P* < 0.05. *P* values derived from analyses of secondary outcomes were unadjusted for multiple comparisons [28]. One participant was excluded from analyses of glucose, insulin, and QUICKI due to the high likelihood of a nonfasted draw at baseline, as evidenced by extremely high values of insulin (187 compared with 13.8 mg/dL at week 16) and glucose (198 compared with 110 mg/dL at week 16). Additionally, this participant’s baseline HbA1c indicated an mean glucose value of ~125 mg/dL over the previous 3 months, further suggesting that the extreme baseline values for glucose and insulin reflect postprandial measures. Removal of these outliers from analyses of glucose, insulin, and QUICKI greatly improved model fit and precision of the estimates.

#### Baseline descriptive characteristics

Descriptive summaries for baseline demographics and study outcomes were generated with the gtsummary [29] package in R and reported as mean ± SD for continuous variables and frequency (*n*, %) for categorical variables.

#### Main effect analyses

The main effect describing the comparative effectiveness of the first-stage interventions (RC compared with HC) was assessed by pooling week 16 outcomes for all participants initially assigned to RC and HC and averaging across any second-stage interventions. All outcomes between RC and HC were then compared using generalized linear models controlling for sex to model the difference of change in scores from baseline to week 16 follow-up [3,21]. The comparative effectiveness of second-stage intervention options (exercise compared with TRE) for nonresponders was assessed using a similar model but rather averaging over for first-stage interventions and excluding any participants identified as responders. All available data were used in regression models. One observation was excluded from analyses of insulin and QUICKI due to a likely data entry error for the insulin value. For each of the statistical methods, the central limit theorem was used to assume approximate normality (i.e., in samples when *n >* 30). A sensitivity analysis for stage 1 and stage 2 was also conducted for the primary and secondary outcomes, in which models were adjusted for baseline values of the outcome in addition to sex.

#### Embedded adaptive interventions analysis

The SMART resulted in 4 embedded adaptive interventions (EAIs) that represent the potential treatment sequences to which a participant could have been assigned. These EAIs are *1*) initiate with a HC diet and, if nonresponder at week 4, augment with 1-on-1 exercise counseling (HC + exercise); *2*) initiate with an HC diet and, if nonresponder at week 4, augment with 1-on-1 TRE counseling (HC+TRE); *3*) initiate with an RC diet and, if nonresponder at week 4, augment with 1-on-1 exercise counseling (RC + exercise), and *4*) initiate with an RC diet and, if nonresponder at week 4, augment with 1-on-1 TRE counseling (RC+TRE).

By design, only nonresponders were rerandomly assigned in the SMART, resulting in responders being overrepresented in a naïve analysis comparing estimates between EAIs. To account for this overrepresentation, an inverse probability of treatment weighted analysis was conducted, in which assigned weights were the inverse of the probability of receiving the given treatment. Therefore, responders received a weight of 2 (i.e., inverse of one-half chance of following any particular sequence) and nonresponders a weight of 4 (i.e., inverse of one-quarter chance). Since observations from responders were consistent with *>*1 EAI, to use the entire sample, the data set was restructured such that there were 2 identical observations per responder. This approach then allowed for reuse of outcomes from responders in estimating the mean outcome of each EAI with a single fitted weighted regression model. Generalized estimating equations were used to compare the change in outcomes from baseline to week 16 of follow-up across the 4 EAI treatment pathways. Lastly, robust standard errors were used to make appropriate inferences with the weighted averages.

### Protocol deviations and adverse events

There were 3 protocol deviations. First, despite reporting no use of weight loss medications, a participant disclosed after enrollment that she had started taking Mounjaro (tirzepatide) before enrollment. The participant was lost to follow-up after being rerandomly assigned to a second-stage intervention assignment and did not attend the week 16 follow-up study visit. Second, a participant was randomly assigned at baseline to the HC group but mistakenly attended the first RC group meeting. Because they already started the RC intervention, the study team decided the participant should continue in the RC group. Third, there were 5 participants who reported adherence to the TRE protocol window was not feasible (i.e., working night shifts, attending social events, and missing family dinner). In these cases, participants and their TRE counselor chose a later 8-h time frame that met their needs. One serious adverse event occurred. A participant was hospitalized after having a stroke and was subsequently withdrawn from the study. The study physician determined that this was not causally related to the diet or exercise intervention or participation in the study.

## Results

Baseline characteristics of study participants by first-stage intervention are presented in Table 1 and by responder status in Supplemental Tables 2 and 3. Eighty-three participants were enrolled into the study (HC, *n* = 41; RC, *n* = 42), and 73 participants completed the 16-wk intervention. Study completers were 53.8 ± 11.7 y (84.3% female, 59% non-Hispanic Black) with obesity (BMI: 38.3 ± 7.2), and data from these participants were used for outcome analyses. At week 4, 36 (43.4% of week 4 completers) participants achieved ≥2.5% weight loss and were identified as responders. Forty-six (55.4% of week 4 completers; *n* = 21 in HC group; *n* = 25 in RC group) participants were identified as nonresponders and were rerandomly assigned to second-stage interventions (TRE: *n* = 22; exercise: *n* = 24).

Weight loss at week 16 was not different between HC and RC diets (0.15 kg; 95% CI: −1.86, 2.16 kg) (Table 2) or TRE and exercise (0.35 kg; 95% CI: −1.72, 2.41 kg) (Table 3). Fasting glucose was reduced more with the HC than that with RC diet (*P* = 0.04) (Table 2), but changes in other measures of glycemic status were not different. No differences in changes of glycemic status were observed between TRE and exercise second-stage strategies (Table 3). Changes in tertiary cardiometabolic outcomes were also not different between HC and RC (Table 4) or TRE and exercise (Table 5). Despite augmenting behavioral weight loss with TRE or exercise counseling, nonresponders achieved less weight loss by the end of the study compared with responders (−2.78 kg; 95% CI: −12.3, 5.90 kg, compared with −6.22 kg; 95% CI: −24.7, 0.36 kg) (Table 6).

Although main effect analyses showed no difference in weight loss between first-stage or second-stage strategies and nonresponders lost less weight compared with responders overall, the EAI analyses indicate a potential first-stage × second-stage interaction. As shown in Table 7, EAIs with sequences of HC+TRE and RC + exercise resulted in ~8-kg weight loss compared with ~2.5 kg weight loss in the EAIs with HC + exercise and RC+TRE. Improvements in glycemic status (Supplemental Table 4) and cardiometabolic health (Supplemental Table 5) were generally greater in the EAIs with greater weight loss. For example, the EAI with HC+TRE produced the greatest reductions in TC, LDL-cholesterol, insulin, and QUICKI. However, the EAI with RC+TRE showed the greatest improvements for SBP and DBP even in the absence of clinically significant weight loss. Sensitivity analyses for stage 1 and stage 2 outcomes, in which models were adjusted for baseline values in addition to sex, yielded results similar to analyses adjusted only for sex (Supplemental Tables 6–8).

## Discussion

This study investigated which combination and sequence of diet plan (RC compared with HC), TRE, and/or exercise is most effective for weight loss and improving glycemic status in people with prediabetes. Contrary to our hypothesis that the RC diet would be more effective for weight loss and improving glycemic status, results showed no difference between diets for weight loss, and the HC diet was more effective for reducing fasting glucose. Both the HC and RC diets were calorie restricted, which resulted in mean weight loss at the accepted threshold for clinical meaningfulness (~5% reduction). We also hypothesized that the addition of exercise counseling would be more beneficial for weight loss and glycemic status than TRE counseling. No statistical differences between exercise and TRE were found for the main effect of second-stage interventions analysis. However, preliminary comparisons among EAIs indicated the effectiveness of second-stage augmentation strategies may be dependent on the diet plan. In this study, augmenting the RC plan with exercise resulted in greater weight loss than augmenting with TRE, but TRE was the more effective augmentation strategy for nonresponders assigned to the HC plan. Note that this study was not statistically powered to detect differences among the 4 EAIs so the quantitative differences reported in this study should be considered as preliminary findings that require further investigation in a fully powered trial.

The carbohydrate–insulin model of obesity posits that insulin secretion in response to carbohydrate intake inhibits lipolysis and promotes fat deposition and weight gain [30]. Therefore, restricting dietary carbohydrate is often promoted as an effective weight loss strategy (especially for people with impaired glycemic status), and there is meta-analytical evidence to support this approach [31–33]. However, the optimal level of carbohydrate restriction is unknown and definitions of what constitutes an RC diet varies considerably [31–34]. Similarly, there is no standard definition of a HC diet [31–34] other than comprising more dietary carbohydrate than the comparator RC diet [35]. Despite the evidence in favor of reducing carbohydrate and energy intake for weight loss, adherence can be difficult in the long term (*>*6 mo) and becomes more difficult with greater carbohydrate restriction [36,37]. The dietary approaches used in this study used an RC prescription of 35% energy from carbohydrates and 50% energy from carbohydrate in the HC diet. These diet plans reflect the higher end of typical RC definitions and lower end of HC definitions, respectively. Therefore, it is possible that actual carbohydrate intakes may not have differed substantially between groups given the relatively modest differences in prescribed carbohydrate intakes and that adherence to counseling-based dietary interventions is typically imperfect. It should also be noted that both diets prescribed identical energy restriction, which is the primary driver of the weight loss [38].

Although glycemic load was not quantified in this study, meal plans for the RC and HC diets emphasized low glycemic load foods such as whole grains, legumes, and nonstarchy vegetables. It is possible that despite the HC diet having a higher overall quantity of carbohydrates the HC diet also provided more fiber, which may at least partially explain the greater reduction in fasting glucose with the HC than that with RC diet [39]. This finding should be replicated and confirmed in additional trials that are designed and powered to test this hypothesis and that include robust measures of dietary adherence. In the present study, A1c was similarly reduced with both diets, although the mean reduction was <0.5%, which is commonly cited as a clinically important reduction in A1c [40]. This is likely a result of the intervention duration of 16 wk (~4 mo). A1c values are representative of mean glucose over the previous 3 mo, so it is possible that glucose concentrations were not meaningfully reduced in participants until later in the intervention period after more weight had been lost. Altogether, results from this study support consuming an overall healthy and energy-restricted diet that emphasizes low-glycemic/high fiber foods for weight loss and improving glycemic status in people with prediabetes.

Among nonresponders to initial dietary interventions, achieved weight loss at week 16 was approximately half of achieved weight loss among responders and was also below the 5% threshold for clinically meaningful weight loss. These findings suggest that the second-stage intervention strategies used in this study may not have been sufficiently effective adaptation strategies. Given that nonresponders lost over 2 kg between weeks 4 and 16, it is possible that greater weight loss could be achieved with a longer duration trial. In addition to augmentation with either exercise or TRE, the premise for selecting these second-stage interventions was that the additional 1-on-1 counseling would supplement the group-based foundational program to promote greater adherence to all intervention components. The lack of meaningful improvements resulting from the counseling-based second-stage interventions suggests that future research may need to use more intensive, and therefore more costly, adaptation strategies such as providing medically tailored meals or groceries and/or providing direct exercise supervision. Future research should also explore why TRE appears to enhance the effects of the HC diet more effectively than exercise, whereas exercise seems to yield greater benefits than TRE when combined with the RC diet. As mentioned earlier, this study was not statistically powered to detect differences among EAIs so these results should be considered as preliminary.

This study had several limitations. First, adherence to the prescribed diets was not rigorously monitored. Although participants were asked to complete 3-d paper food logs at baseline and week 16 to assess dietary adherence, compliance with this task was poor, and the resulting data were insufficient for meaningful analysis. It is possible that participants of both diets ate more or less than their prescribed carbohydrate amount, which would affect observed changes in study outcomes. The results of this study should therefore be interpreted as counseling to follow an HC or RC diet rather than consuming those dietary patterns, which cannot be definitively known in this study. Second, this study had a relatively small sample size and was powered to detect a medium effect size for the difference in weight loss between HC and RC diets. Related, the observed nonresponse rate of ~55% was less than the anticipated 67% nonresponse rate, which reduced the number of nonresponders assigned to exercise or TRE and further reduced achieved statistical power for the main effect of second-stage interventions comparison. Finally, the majority of participants were (53.8 ± 11.7 y) females (84.3%), which may reduce the generalizability of findings to males. However, analyses controlled for sex and showed no effect.

This study also had several strengths. First, the novel SMART design permits investigation into the effects of treatment sequence, which may ultimately translate to more robust clinical treatments for people with obesity and prediabetes. Second, the 1-on-1 exercise or TRE counseling sessions allow for more personalized regimens, increasing the likelihood of dietary adherence and participant satisfaction. Third, all participants received group-based counseling sessions, which are shown to be more effective than individual treatments for weight management [41].

In conclusion, results from this SMART indicate that a group-based weight loss intervention was effective for reducing body weight and improving A1c using both calorie-restricted HC and RC diet prescriptions and that the HC diet may reduce fasting glucose more than RC. No significant differences were observed among A1c, body fat, SBP, DBP, TC, HDL-cholesterol, LDL-cholesterol, triglycerides, insulin, and QUICKI. However, individuals with obesity and prediabetes who are unable to achieve early weight loss of ≥2.5% may require more intensive and costly intervention strategies (i.e., meal provisions and supervised exercise) to improve obesity treatment outcomes.

## Supplementary Material

Appendix A.

## Figures and Tables

**TABLE 1:** Overall and first-stage intervention baseline participant characteristics.

Characteristic	Overall	HC	RC
			
	*N* = 83	*n* = 41	*n* = 42

Age (y)	53.8 ± 11.7	53.2 ± 11.7	54.3 ± 11.9
Sex			
Female	70 (84.3)	34 (82.9)	36 (85.7)
Male	13 (15.7)	7 (17.1)	6 (14.3)
Unknown	0	0	0
Race			
Asian	3 (3.61)	2 (4.88)	1 (2.38)
Black or African American	49 (59.0)	26 (63.4)	23 (54.8)
White	26 (31.3)	12 (29.3)	14 (33.3)
Other	3 (3.61)	1 (2.44)	2 (4.76)
Unknown	2 (2.41)	0	2 (4.76)
Ethnicity			
Hispanic	2 (2.41)	0	2 (4.76)
Non-Hispanic	74 (89.2)	36 (87.8)	38 (90.5)
Unknown	7 (8.43)	5 (12.2)	2 (4.76)
Body weight (kg)	104 ± 23.0	104 ± 22.1	104 ± 24.1
Height (cm)	64.8 ± 2.98	64.9 ± 3.12	64.7 ± 2.87
BMI	38.2 ± 7.49	38.3 ± 7.00	38.0 ± 8.02
WC (cm)	116 ± 14.6	117 ± 13.2	116 ± 16.0
SBP (mm Hg)	127 ± 14.9	125 ± 12.8	129 ± 16.6
DBP (mm Hg)	81.5 ± 9.12	80.7 ± 7.08	82.3 ± 10.7
Fasting glucose (mg/dL)	92.3 ± 19.2	95.2 ± 23.3	89.4 ± 13.6
Fasting insulin (U/mL)	12.7 ± 7.68	13.0 ± 6.51	12.5 ± 8.75
QUICKI	0.338 ± 0.031	0.334 ± 0.030	0.342 ± 0.032
A1c (%)	5.93 ± 0.295	6.00 ± 0.281	5.87 ± 0.295
TC (mg/dL)	182 ± 40.4	183 ± 39.4	181 ± 41.9
HDL (mg/dL)	54.2 ± 16.8	52.9 ± 16.9	55.4 ± 16.9
LDL (mg/dL)	112 ± 34.1	113 ± 32.2	110 ± 36.2
TG (mg/dL)	103 ± 40.9	105 ± 46.1	100 ± 35.5

Abbreviations: A1c, hemoglobin A1c; DBP, diastolic blood pressure; HC, high-carbohydrate diet; HDL, high-density lipoprotein; LDL, low density lipoprotein; QUICKI, quantitative insulin-sensitivity check index; RC, reduced carbohydrate diet; SBP, systolic blood pressure; TC, total cholesterol; TG, triglyceride; WC, waist circumference.

**TABLE 2:** Within-group and between-group differences for weight loss (primary outcome) and glycemic status (secondary outcomes) by first-stage intervention assignments.

Outcome	High-carbohydrate diet change (*n* = 41), mean (95% CI)	Reduced carbohydrate change (*n* = 42), mean (95% CI)	Between-group differences, mean (95% CI)

Weight (kg)	−5.26 (−6.93, −3.59)	−5.41 (−7.27, −3.59)	0.15 (−1.86, 2.16)
Hemoglobin A1c (%)	−0.17 (−0.28, −0.06)	−0.16 (−0.27, −0.04)	−0.02 (−0.14, 0.11)
Glucose (mg/dL)	−8.86 (−14.29, −3.44)	−2.45 (−8.13, 3.22)	−6.41 (−12.40, −0.42)
Insulin (U/mL)	−2.63 (−5.21, −0.04)	−1.90 (−4.6, 0.8)	−0.72 (−3.60, 2.15)
QUICKI	0.009 (−0.002, 0.020)	0.007 (−0.004, 0.018)	0.002 (−0.010, 0.013)

Abbreviation: QUICKI, quantitative insulin-sensitivity check index.

Changes in first-stage interventions from baseline to week 16 were compared using generalized linear models controlling for sex.

**TABLE 3:** Within-group and between-group differences for weight loss (primary outcome) and glycemic status (secondary outcomes) by second-stage intervention assignments.

Outcome	Exercise (*n* =21), mean (95% CI)	Time restricted eating (*n* =20), mean (95% CI)	Between-group differences, mean (95% CI)

Weight (kg)	−3.89 (−6.04, −1.74)	−4.24 (−6.54, −1.93)	0.35 (−1.72, 2.41)
Hemoglobin A1c (%)	−0.12 (−0.3, 0.06)	−0.26 (−0.45, −0.06)	0.14 (−0.04, 0.31)
Glucose (mg/dL)	−1.70 (−11.57, 8.16)	−9.75 (−20.35, 0.84)	8.05 (−1.41, 17.51)
Insulin (U/mL)	−0.46 (−4.68, 3.75)	−0.50 (−5.06, 4.06)	0.04 (−4.05, 4.13)
QUICKI	−0.001 (−0.02, 0.015)	0.007 (−0.01, 0.025)	−0.009 (−0.02, 0.01)

Abbreviation: QUICKI, quantitative insulin-sensitivity check index.

Changes in second-stage interventions from baseline to week 16 were compared in nonresponders with generalized linear models controlling for sex and were averaged over first-stage interventions.

**TABLE 4:** Within-group and between-group differences for cardiometabolic measures (tertiary outcomes) by first-stage intervention assignments.

Outcome	High-carbohydrate diet change (*n* =41), mean (95% CI)	Reduced carbohydrate change (*n* =42), mean (95% CI)	Between-group differences, mean (95% CI)

BF (kg)	−2.13 (−3.19, −1.06)	−2.62 (−3.80, −1.44)	0.50 (−0.79, 1.78)
SBP (mm Hg)	−1.61 (−6.86, 3.63)	−8.45 (−14.25, −2.66)	6.84 (0.51, 13.17)
DBP (mm Hg)	−1.14 (−4.71, 2.43)	−4.60 (−8.55, −0.66)	3.46 (−0.85, 7.77)
TC (mg/dL)	−8.26 (−16.28, −0.24)	−1.18 (−9.81, 7.45)	−7.08 (−16.36, 2.21)
HDL (mg/dL)	−0.87 (−5.52, 3.77)	−4.71 (−10.08, 0.67)	3.84 (−1.83, 9.50)
LDL (mg/dL)	−6.51 (−13.19, 0.17)	−2.21 (−9.40, 4.99)	−4.30 (−12.04, 3.43)
TG (mg/dL)	−2.02 (−16.13, 12.10)	−2.34 (−17.53, 12.85)	0.32 (−16.02, 16.66)

Changes in first-stage interventions from baseline to week 16 were compared using generalized linear models controlling for sex.

Abbreviations: BF, body fat; DBP, diastolic blood pressure; HDL, high-density lipoprotein; LDL, low density lipoprotein; SBP, systolic blood pressure; TC, total cholesterol; TG, triglyceride.

**TABLE 5:** Within-group and between-group differences for cardiometabolic measures (tertiary outcomes) by second-stage intervention assignments.

Outcome	Exercise (*n* =21), mean (95% CI)	Time restricted eating (*n* =20), mean (95% CI)	Between-group differences, mean (95% CI)

BF (kg)	−1.12 (−2.57, 0.33)	−1.57 (−3.13, −0.02)	0.45 (−0.94, 1.84)
SBP (mm Hg)	−5.70 (−16.13, 4.74)	−4.40 (−15.56, 6.75)	−1.29 (−11.37, 8.79)
DBP (mm Hg)	−2.14 (−9.09, 4.82)	−2.80 (−10.24, 4.63)	0.66 (−6.05, 7.38)
TC (mg/dL)	3.69 (−7.61, 14.99)	−3.82 (−15.95, 8.31)	7.51 (−3.32, 18.34)
HDL (mg/dL)	0.37 (−8.55, 9.30)	−5.53 (−15.21, 4.15)	5.90 (−2.93, 14.74)
LDL (mg/dL)	1.50 (−9.17, 12.16)	−0.19 (−11.64, 11.26)	1.69 (−8.54, 11.91)
TG (mg/dL)	−18.38 (−39.31, 2.54)	−14.52 (−36.98, 7.95)	−3.87 (−23.93, 16.19)

Changes in second-stage interventions from baseline to week 16 were compared in nonresponders only with generalized linear models controlling for sex and were averaged over first-stage interventions.

Abbreviations: BF, body fat; CI, confidence interval; DBP, diastolic blood pressure; HDL, high-density lipoprotein; LDL, low density lipoprotein; SBP, systolic blood pressure; TC, total cholesterol; TG, triglyceride.

**TABLE 6:** Weight loss by responder status.

Outcome	Nonresponders (*n* = 41)	Responders (*n* = 32)	*P* ^1^

Change week 4 body weight (kg)			<0.001
Mean (SD)	−0.571 (1.43)	−3.79 (1.30)	
Median (minimum, maximum)	−0.907 (−3.54, 2.54)	−3.63 (−6.99, −1.81)	
Change week 16 body weight (kg)			<0.001
Mean (SD)	−2.78 (3.49)	−6.22 (4.87)	
Median (minimum, maximum)	−2.63 (−12.3, 5.90)	−4.63 (−24.7, 0.363)	

Abbreviation: SD, standard deviation.

1 Wilcoxon rank sum test.

**TABLE 7:** Comparison of weight loss for embedded adaptive interventions (*n* = 82).

Embedded AI	First-stage intervention	Status	Second-stage intervention	ΔWeight (kg), mean (95% CI)

1	High-carbohydrate diet	Responder	Control with HC diet	−2.30 (−4.46, −0.14)
		Nonresponder	+ Exercise	
2	High-carbohydrate diet	Responder	Control with HC diet	−8.25 (−12.44, −4.06)
		Nonresponder	+ Time restricted eating	
3	Reduced carbohydrate diet	Responder	Control with RC diet	−8.15 (−12.6, −3.7)
		Nonresponder	+ Exercise	
4	High-carbohydrate diet counseling	Responder	Cont. with RC diet	−2.65 (−5.15, −0.16)
		Nonresponder	+ Time restricted eating	

Weighted and replicated regression was used to rank order the change in outcomes from baseline to week 16 follow-up across the 4 embedded adaptive intervention pathways.

## Data Availability

Data described in the manuscript, code book, and analytic code will be made available upon request to the corresponding author.

## Appendix A. Supplementary data

Supplementary data to this article can be found online at https://doi.org/10.1016/j.ajcnut.2025.07.034.

## Abbreviations:

A1c: hemoglobin A1c
AF: activity factor
AMR: analytical measurement range
DBP: diastolic blood pressure
DPP: Diabetes Prevention Program
EAI: embedded adaptive interventions
HC: higher carbohydrate
QUICKI: quantitative insulin-sensitivity check index
RC: reduced carbohydrate
SBP: systolic blood pressure
SMART: Sequential Multiple Assignment Randomized Trial
TC: total cholesterol
TRE: time restricted eating
UAB: University of Alabama at Birmingham

## References

[1] MacLean PS, Rothman AJ, Nicastro HL, Czajkowski SM, Agurs-Collins T, Rice EL, , The Accumulating Data to Optimally Predict Obesity Treatment (ADOPT) core measures project: rationale and approach, Obesity (Silver Spring). 26 (Suppl 2) (2018) S6–S15. 29575780 10.1002/oby.22154 PMC5973529

[2] MacLean PS, Wing RR, Davidson T, Epstein L, Goodpaster B, Hall KD, , NIH working group report: innovative research to improve maintenance of weight loss, Obesity (Silver Spring) 23 (1) (2015) 7–15. 25469998 10.1002/oby.20967 PMC5841916

[3] Almirall D, Nahum-Shani I, Sherwood NE, Murphy SA, Introduction to SMART designs for the development of adaptive interventions: with application to weight loss research, Transl, Behav. Med. 4 (3) (2014) 260–274. 25264466 10.1007/s13142-014-0265-0 PMC4167891

[4] Diabetes Prevention Program (DPP) Research Group, The Diabetes Prevention Program (DPP): description of lifestyle intervention, Diabetes Care 25 (12) (2002) 2165–2171. 12453955 10.2337/diacare.25.12.2165 PMC1282458

[5] Knowler WC, Barrett-Connor E, Fowler SE, Hamman RF, Lachin JM, Walker EA, , Reduction in the incidence of type 2 diabetes with lifestyle intervention or metformin, N. Engl. J. Med. 346 (6) (2002) 393–403. 11832527 10.1056/NEJMoa012512 PMC1370926

[6] Hjorth MF, Bray GA, Zohar Y, Urban L, Miketinas DC, Williamson DA, , Pretreatment fasting glucose and insulin as determinants of weight loss on diets varying in macronutrients and dietary fibers—the POUNDS LOST study, Nutrients 11 (3) (2019) 586. 30861997 10.3390/nu11030586 PMC6470525

[7] Cornier MA, Donahoo WT, Pereira R, Gurevich I, Westergren R, Enerback S, , Insulin sensitivity determines the effectiveness of dietary macronutrient composition on weight loss in obese women, Obes. Res. 13 (4) (2005) 703–709. 15897479 10.1038/oby.2005.79

[8] Pittas AG, Das SK, Hajduk CL, Golden J, Saltzman E, Stark PC, , A low-glycemic load diet facilitates greater weight loss in overweight adults with high insulin secretion but not in overweight adults with low insulin secretion in the CALERIE Trial, Diabetes Care 28 (12) (2005) 2939–2941. 16306558 10.2337/diacare.28.12.2939

[9] EllisonKM, WyattHR, HillJO, SayerRD, Should carbohydrate-modified diets be the first option for weight loss in people with impaired glucose metabolism? A scoping review, Obes. Rev. 25 (2024) e13706.38355200 10.1111/obr.13706

[10] Ebbeling CB, Leidig MM, Feldman HA, Lovesky MM, Ludwig DS, Effects of a low-glycemic load vs low-fat diet in obese young adults: a randomized trial, JAMA 297 (19) (2007) 2092–2102. 17507345 10.1001/jama.297.19.2092

[11] Ehrlicher SE, Chui TK, Clina JG, Ellison KM, Sayer RD, The data behind popular diets for weight loss, Med. Clin. North Am 106 (5) (2022) 739–766. 36154698 10.1016/j.mcna.2022.05.003

[12] Cienfuegos S, McStay M, Gabel K, Varady KA, Time restricted eating for the prevention of type 2 diabetes, J. Physiol. 600 (5) (2022) 1253–1264. 34418079 10.1113/JP281101

[13] Jamshed H, Steger FL, Bryan DR, Richman JS, Warriner AH, Hanick CJ, , Effectiveness of early time-restricted eating for weight loss, fat loss, and cardiometabolic health in adults with obesity: a randomized clinical trial, JAMA Intern. Med. 182 (9) (2022) 953–962. 35939311 10.1001/jamainternmed.2022.3050 PMC9361187

[14] Steger FL, Jamshed H, Bryan DR, Richman JS, Warriner AH, Hanick CJ, , Early time-restricted eating affects weight, metabolic health, mood, and sleep in adherent completers: a secondary analysis, Obesity (Silver Spring) 31 (S1) (2023) 96–107. 10.1002/oby.23614 PMC9877132 36518092

[15] Falci SG, Marques LS, CONSORT: when and how to use it, Dental Press J. Orthod. 20 (3) (2015) 13–15. 26154451 10.1590/2176-9451.20.3.013-015.ebo PMC4520133

[16] Sayer RD, Speaker KJ, Pan Z, Peters JC, Wyatt HR, Hill JO, Equivalent reductions in body weight during the Beef WISE Study: beef’s role in weight improvement, satisfaction and energy, Obes, Sci. Pract. 3 (3) (2017) 298–310. 29071106 10.1002/osp4.118 PMC5598025

[17] Stevens J, Truesdale KP, McClain JE, Cai J, The definition of weight maintenance, Int. J. Obes. (Lond). 30 (3) (2006) 391–399. 16302013 10.1038/sj.ijo.0803175

[18] Moyer JC, Li F, Cook AJ, Heagerty PJ, Pals SL, Turner EL, , Evaluating analytic models for individually randomized group treatment trials with complex clustering in nested and crossed designs, Stat. Med. 43 (25) (2024) 4796–4818. 39225281 10.1002/sim.10206

[19] Centers for Disease Control and Prevention, National Diabetes Prevention Program [Internet], 2023. Available from: https://www.cdc.gov/diabetes/prevention/index.html.

[20] Frankenfield D, Roth-Yousey L, Compher C, Comparison of predictive equations for resting metabolic rate in healthy nonobese and obese adults: a systematic review, J. Am. Diet. Assoc. 105 (5) (2005) 775–789. 15883556 10.1016/j.jada.2005.02.005

[21] American Diabetes Association, The diabetic exchange list (exchange diet) [Internet]. Available from: https://www.diabetesed.net/page/_files/THE-DIABETIC-EXCHANGE-LIST.pdf.

[22] Sherwood NE, Crain AL, Seburg EM, Butryn ML, Forman EM, Crane MM, , BestFIT sequential multiple assignment randomized trial results: a SMART approach to developing individualized weight loss treatment sequences, Ann. Behav. Med. 56 (3) (2022) 291–304. 34415011 10.1093/abm/kaab061 PMC8887581

[23] Chen H, Sullivan G, Quon MJ, Assessing the predictive accuracy of QUICKI as a surrogate index for insulin sensitivity using a calibration model, Diabetes 54 (7) (2005) 1914–1925. 15983190 10.2337/diabetes.54.7.1914

[24] Stein MW, Clinical methods of enzymatic analysis, Academic Press, Cambridge, Massachusetts, 1965.

[25] Harris PA, Taylor R, Minor BL, Elliott V, Fernandez M, O’Neal L, , The REDCap consortium: building an international community of software platform partners, J. Biomed. Inform. 95 (2019) 103208. 31078660 10.1016/j.jbi.2019.103208 PMC7254481

[26] Harris PA, Taylor R, Thielke R, Payne J, Gonzalez N, Conde JG, Research electronic data capture (REDCap)—a metadata-driven methodology and workflow process for providing translational research informatics support, J. Biomed. Inform. 42 (2) (2009) 377–381. 18929686 10.1016/j.jbi.2008.08.010 PMC2700030

[27] R Foundation, R: a language and environment for statistical computing, R Foundation for Statistical Computing, Vienna, Austria, 2024.

[28] Feise RJ, Do multiple outcome measures require p-value adjustment? BMC Med. Res. Methodol. 2 (2002) 8. 12069695 10.1186/1471-2288-2-8 PMC117123

[29] Sjoberg DD, Whiting K, Curry M, Lavery JA, Larmarange J, Reproducible summary tables with the gtsummary package, R J 13 (1) (2021) 570–580.

[30] Ludwig DS, Majzoub JA, Al-Zahrani A, Dallal GE, Blanco I, Roberts SB, High glycemic index foods, overeating, and obesity, Pediatrics 103 (3) (1999) E26. 10049982 10.1542/peds.103.3.e26

[31] Mansoor N, Vinknes KJ, Veierød MB, Retterstøl K, Effects of low-carbohydrate diets v. low-fat diets on body weight and cardiovascular risk factors: a meta-analysis of randomised controlled trials, Br. J. Nutr. 115 (3) (2016) 466–479. 26768850 10.1017/S0007114515004699

[32] Sackner-Bernstein J, Kanter D, Kaul S, Dietary intervention for overweight and obese adults: comparison of low-carbohydrate and low-fat diets, A meta-analysis, PLoS One 10 (10) (2015) e0139817. 26485706 10.1371/journal.pone.0139817 PMC4618935

[33] Nordmann AJ, Nordmann A, Briel M, Keller U, Yancy WS Jr., Brehm BJ, , Effects of low-carbohydrate vs low-fat diets on weight loss and cardiovascular risk factors: a meta-analysis of randomized controlled trials, Arch. Intern. Med. 166 (3) (2006) 285–293. 16476868 10.1001/archinte.166.3.285

[34] Hu T, Mills KT, Yao L, Demanelis K, Eloustaz M, Yancy WS Jr., , Effects of low-carbohydrate diets versus low-fat diets on metabolic risk factors: a meta-analysis of randomized controlled clinical trials, Am. J. Epidemiol. 176 (Suppl 7) (2012) S44–S54. 23035144 10.1093/aje/kws264 PMC3530364

[35] Jung CH, Choi KM, Impact of high-carbohydrate diet on metabolic parameters in patients with type 2 diabetes, Nutrients 9 (4) (2017) 322. 28338608 10.3390/nu9040322 PMC5409661

[36] Sacks FM, Bray GA, Carey VJ, Smith SR, Ryan DH, Anton SD, , Comparison of weight-loss diets with different compositions of fat, protein, and carbohydrates, N. Engl. J. Med. 360 (9) (2009) 859–873. 19246357 10.1056/NEJMoa0804748 PMC2763382

[37] Kumar NK, Merrill JD, Carlson S, German J, Yancy WS Jr., Adherence to low-carbohydrate diets in patients with diabetes: a narrative review, Diabetes Metab. Syndr. Obes. 15 (2022) 477–498. 35210797 10.2147/DMSO.S292742 PMC8863186

[38] Redman LM, Ravussin E, Caloric restriction in humans: impact on physiological, psychological, and behavioral outcomes, Antioxid. Redox. Signal. 14 (2) (2011) 275–287. 20518700 10.1089/ars.2010.3253 PMC3014770

[39] McRae MP, Dietary fiber intake and type 2 diabetes mellitus: an umbrella review of meta-analyses, J. Chiropr. Med. 17 (1) (2018) 44–53. 29628808 10.1016/j.jcm.2017.11.002 PMC5883628

[40] Little RR, Rohlfing CL, The long and winding road to optimal HbA1c measurement, Clin. Chim. Acta. 418 (2013) 63–71. 23318564 10.1016/j.cca.2012.12.026 PMC4762213

[41] Paul-Ebhohimhen V, Avenell A, A systematic review of the effectiveness of group versus individual treatments for adult obesity, Obes. Facts. 2 (1) (2009) 17–24. 20054200 10.1159/000186144 PMC6444750

