# Supplementary material for: Adaptive dietary and exercise strategies for weight loss in Adults with Prediabetes Trial (ADAPT): a sequential multiple assignment randomized trial
Source: Am J Clin Nutr. Author manuscript; Available in PMC 2026 Apr 15. (PMC12674035; doi:10.1016/j.ajcnut.2025.07.034)

Supplemental Material

*Adaptive Dietary and exercise strategies for weight loss in Adults with Prediabetes Trial (ADAPT): A Sequential Multiple Assignment Randomized Trial*

Katie M. Ellison, PhD, RDN et al.

**Supplemental Table 1.** Weight management program topics.

| 16-Week Weight Loss Phase | Session Topic |
| --- | --- |
| Week 1 | Getting Set for Success |
| Week 2 | What is Energy Balance? |
| Week 3 | Taking Control of Your Eating |
| Week 4 | Keep Your Heart Healthy |
| Week 5 | Facing Frustration |
| Week 6 | Effective Problem Solving |
| Week 7 | Social Support |
| Week 8 | Talking Back to Negative Thoughts |
| Week 9 | Managing Stress |
| Week 10 | Coping with High-Risk Situations |
| Week 11 | Positive Body Image |
| Week 12 | Staying Motivated |
| Week 13 | Emotions & You |
| Week 14 | Strategies for Eating Out |
| Week 15 | Strategies for Success |
| Week 16 | Relapse Prevention |

**Supplemental Table 2.** Overall and 1^st^ stage intervention baseline participant characteristics by response status.

|  | Mean ± SD^1^ or *n* (%) | | | | | |
| --- | --- | --- | --- | --- | --- | --- |
| Characteristic | Overall | | HC^1^ | | RC^1^ | |
|  | Responders | Non-responders | Responders | Non-responders | Responders | Non-responders |
|  | *n* = 36 | *n* = 46 | *n* = 19 | *n* = 21 | *n* = 17 | *n* = 25 |
| Age (yrs) | 56.2 ± 11.4 | 52.2 ± 11.8 | 53.2 ± 11.7 | 53.9 ± 11.9 | 59.5 ± 10.3 | 50.8 ± 11.8 |
| Sex, *n* (%) |  |  |  |  |  |  |
| Female | 27 (75.0) | 42 (91.3) | 14 (73.7) | 19 (90.5) | 13 (76.5) | 23 (92.0) |
| Male | 9 (25.0) | 4 (8.70) | 5 (26.3) | 2 (9.52) | 4 (23.5) | 2 (8.00) |
| Unknown | 0 | 0 | 0 | 0 | 0 | 0 |
| Race, *n* (%) |  |  |  |  |  |  |
| Asian | 2 (5.56) | 1 (2.17) | 2 (10.5) | 0 | 0 | 1 (4.00) |
| Black or African American | 16 (44.4) | 33 (71.7) | 11 (57.9) | 15 (71.4) | 5 (29.4) | 18 (72.0) |
| White | 15 (41.7) | 11 (23.9) | 6 (31.6) | 6 (28.6) | 9 (52.9) | 5 (20.0) |
| Other | 2 (5.56) | 0 | 0 | 0 | 2 (11.8) | 0 |
| Unknown | 1 (2.78) | 1 (2.17) | 0 | 0 | 1 (5.88) | 1 (4.00) |
| Ethnicity, *n* (%) |  |  |  |  |  |  |
| Hispanic | 2 (5.56) | 0 | 0 | 0 | 2 (11.8) | 0 |
| Non-Hispanic | 30 (83.3) | 43 (93.5) | 16 (84.2) | 19 (90.5) | 14 (82.4) | 24 (96.0) |
| Unknown | 4 (11.1) | 3 (6.52) | 3 (15.8) | 2 (9.52) | 1 (5.88) | 1 (4.00) |
| Body wt^1^ (kg) | 101 ± 20.7 | 106 ± 24.8 | 99.2 ± 16.5 | 109 ± 26.1 | 103 ± 24.9 | 104 ± 24.0 |
| Height (cm) | 65.0 ± 3.17 | 64.7 ± 2.88 | 65.1 ± 3.35 | 64.8 ± 3.04 | 64.9 ± 3.05 | 64.6 ± 2.80 |
| BMI^1^ (kg/m^2^) | 37.2 ± 7.17 | 38.9 ± 7.77 | 36.5 ± 5.98 | 39.8 ± 7.71 | 37.9 ± 8.43 | 38.1 ± 7.90 |
| WC^1^ (cm) | 115 ± 14.6 | 117 ± 14.8 | 113 ± 11.9 | 119 ± 14.2 | 116 ± 17.5 | 115 ± 15.3 |
| SBP^1^ (mm Hg) | 129 ± 16.2 | 126 ± 13.8 | 127 ± 14.9 | 123 ± 10.7 | 131 ± 17.8 | 127 ± 15.9 |
| DBP^1^ (mm Hg) | 81.5 ± 8.18 | 81.4 ± 9.99 | 81.4 ± 8.46 | 79.8 ± 5.78 | 81.6 ± 8.12 | 82.7 ± 12.4 |
| Fasting glucose (mg/dL) | 89.9 ± 21.6 | 94.2 ± 17.2 | 94.4 ± 27.6 | 96.1 ± 20.1 | 84.8 ± 10.7 | 92.6 ± 14.6 |
| Fasting insulin (U/mL) | 11.6 ± 6.18 | 12.4 ± 6.38 | 13.4 ± 6.47 | 11.9 ± 6.03 | 9.47 ± 5.22 | 12.8 ± 6.77 |
| QUICKI^1^ | 0.343 ± 0.033 | 0.335 ± 0.029 | 0.334 ± 0.030 | 0.336 ± 0.030 | 0.354 ± 0.033 | 0.335 ± 0.029 |
| A1c^1^ (%) | 5.86 ± 0.307 | 5.98 ± 0.273 | 5.95 ± 0.261 | 6.04 ± 0.298 | 5.76 ± 0.332 | 5.94 ± 0.248 |
| TC^1^ (mg/dL) | 191 ± 43.4 | 175 ± 37.3 | 188 ± 39.6 | 178 ± 40.5 | 195 ± 48.3 | 172 ± 35.0 |
| HDL-C^1^ (mg/dL) | 54.3 ± 18.2 | 54.1 ± 16.1 | 50.9 ± 13.9 | 54.7 ± 19.8 | 57.9 ± 22.0 | 53.6 ± 12.6 |
| LDL-C^1^ (mg/dL) | 119 ± 36.1 | 106 ± 32.0 | 119 ± 32.8 | 107 ± 32.2 | 120 ± 40.5 | 104 ± 32.3 |
| TG^1^ (mg/dL) | 110 ± 40.2 | 97.6 ± 41.5 | 110 ± 40.5 | 101 ± 52.1 | 108 ± 41.1 | 94.6 ± 30.7 |

^1^ A1c, hemoglobin A1c; BMI, body mass index; DBP, diastolic blood pressure; HC, high carbohydrate diet; HDL-C, high-density lipoprotein cholesterol; LDL-C, low-density lipoprotein cholesterol; QUICKI, quantitative insulin-sensitivity check index; RC, reduced carbohydrate diet; SBP, systolic blood pressure; SD, standard deviation; TC, total cholesterol; TG, triglycerides; WC, waist circumference; wt, weight.

**Supplemental Table 3.** Overall and 2nd stage intervention baseline participant characteristics.

|  | Mean ± SD^1^ or *n* (%) | | | | | |
| --- | --- | --- | --- | --- | --- | --- |
| Characteristic | Overall | | HC^1^ | | RC^1^ | |
|  | TRE^1^ | Exercise | TRE^1^ | Exercise | TRE^1^ | Exercise |
|  | *n* = 22 | *n* = 24 | *n* = 9 | *n* = 12 | *n* = 13 | *n* = 12 |
| Age (yrs) | 49.6 ± 12.2 | 54.5 ± 11.2 | 53.0 ± 14.7 | 54.5 ± 10.1 | 47.3 ± 10.1 | 54.5 ± 12.7 |
| Sex, *n* (%) |  |  |  |  |  |  |
| Female | 21 (95.45) | 21 (87.5) | 8 (88.9) | 11 (91.7) | 13 (100.0) | 10 (83.3) |
| Male | 1 (4.55) | 3 (12.5) | 1 (11.1) | 1 (8.33) | 0 | 2 (16.7) |
| Race, *n* (%) |  |  |  |  |  |  |
| Asian | 0 | 1 (4.17) | 0 | 0 | 0 | 1 (8.33) |
| Black or African American | 16 (72.7) | 17 (70.8) | 6 (66.7) | 9 (75.0) | 10 (76.9) | 8 (66.7) |
| White | 5 (22.7) | 6 (25.0) | 3 (33.3) | 3 (25.0) | 2 (15.4) | 3 (25.0) |
| Other | 0 | 0 | 0 | 0 | 0 | 0 |
| Unknown | 1 (4.55) | 0 | 0 | 0 | 1 (7.69) | 0 |
| Ethnicity, *n* (%) |  |  |  |  |  |  |
| Hispanic | 0 | 0 | 0 | 0 | 0 | 0 |
| Non-Hispanic | 20 (90.9) | 23 (95.8) | 8 (88.9) | 11 (91.7) | 12 (92.3) | 12 (100.0) |
| Unknown | 2 (9.10) | 1 (4.17) | 1 (11.1) | 1 (8.33) | 1 (7.69) | 0 |
| Body wt^1^ (kg) | 104 ± 20.9 | 108 ± 28.2 | 107 ± 17.4 | 110 ± 31.9 | 102 ± 23.5 | 106 ± 25.4 |
| Height (cm) | 64.8 ± 2.59 | 64.6 ± 3.18 | 65.1 ± 2.66 | 64.5 ± 3.39 | 64.6 ± 2.63 | 64.6 ± 3.09 |
| BMI^1^ (kg/m^2^) | 38.2 ± 6.13 | 39.5 ± 9.11 | 39.0 ± 5.08 | 40.4 ± 9.40 | 37.7 ± 6.92 | 38.5 ± 9.13 |
| WC^1^ (cm) | 117 ± 13.2 | 117 ± 16.5 | 119 ± 11.2 | 119 ± 16.7 | 116 ± 14.6 | 115 ± 16.7 |
| SBP^1^ (mm Hg) | 123 ± 15.4 | 128 ± 11.9 | 124 ± 12.5 | 123 ± 9.56 | 122 ± 17.6 | 133 ± 12.5 |
| DBP^1^ (mm Hg) | 81.7 ± 13.5 | 81.2 ± 5.14 | 80.2 ± 6.17 | 79.5 ± 5.72 | 82.7 ± 17.0 | 82.7 ± 4.22 |
| Fasting glucose (mg/dL) | 95.7 ± 22.1 | 92.8 ± 11.3 | 99.9 ± 29.3 | 93.3 ± 9.30 | 92.8 ± 16.2 | 92.3 ± 13.4 |
| Fasting insulin (U/mL) | 12.9 ± 5.99 | 11.9 ± 6.82 | 13.3 ± 4.79 | 10.8 ± 6.82 | 12.6 ± 6.87 | 13.0 ± 6.95 |
| QUICKI^1^ | 0.331 ± 0.026 | 0.339 ± 0.032 | 0.326 ± 0.025 | 0.344 ± 0.032 | 0.331 ± 0.027 | 0.330 ± 0.032 |
| A1c^1^ (%) | 6.06 ± 0.298 | 5.92 ± 0.238 | 6.20 ± 0.312 | 5.93 ± 0.246 | 5.97 ± 0.263 | 5.91 ± 0.239 |
| TC^1^ (mg/dL) | 174 ± 35.4 | 175 ± 39.7 | 170 ± 40.1 | 184 ± 41.5 | 177 ± 33.1 | 167 ± 37.6 |
| HDL-C^1^ (mg/dL) | 55.8 ± 17.3 | 52.6 ± 15.0 | 53.0 ± 20.7 | 56.0 ± 19.8 | 57.7 ± 15.1 | 49.3 ± 7.50 |
| LDL-C^1^ (mg/dL) | 101 ± 27.6 | 110 ± 35.6 | 99.4 ± 26.0 | 113 ± 36.2 | 103 ± 29.7 | 106 ± 36.3 |
| TG^1^ (mg/dL) | 102 ± 34.6 | 93.9 ± 47.3 | 106 ± 34.4 | 97.7 ± 63.6 | 98.8 ± 35.9 | 90.1 ± 24.7 |

^1^ A1c, hemoglobin A1c; BMI, body mass index; DBP, diastolic blood pressure; HC, high carbohydrate diet; HDL-C, high-density lipoprotein cholesterol; LDL-C, low-density lipoprotein cholesterol; QUICKI, quantitative insulin-sensitivity check index; RC, reduced carbohydrate diet; SBP, systolic blood pressure; SD, standard deviation; TC, total cholesterol; TG, triglycerides; TRE, time restricted eating; WC, waist circumference; wt, weight.

**Supplemental Table 4.** Estimates for adaptive intervention groups for glycemic-related outcomes (*n* =82).

| Embedded adaptive intervention^1^ | Outcome | Estimated Mean^2^ | CI^1^ | P |
| --- | --- | --- | --- | --- |
| HC + TRE | A1c^1^ (%) | -0.280 | (-0.53, -0.04) | 0.0.250 |
| HC + Ex |  | -0.07 | (-0.22, 0.08) | 0.3509 |
| RC + TRE |  | -0.13 | (-0.28, 0.03) | 0.1137 |
| RC + Ex |  | -0.18 | (-0.45, 0.08) | 0.1789 |
| HC + TRE | Glucose (mg/dL) | -6.534 | (-13.21, 0.14) | 0.0551 |
| HC + Ex |  | -11.943 | (-18.27, -5.62) | 0.0002 |
| RC + TRE |  | -10.543 | (-16.58, -4.5) | 0.0006 |
| RC + Ex |  | 5.909 | (-3.07, 14.89) | 0.1971 |
| HC + TRE | Insulin (U/mL) | -4.76 | (-13.5, 3.98) | 0.2854 |
| HC + Ex |  | -0.52 | (-4.78, 3.75) | 0.8128 |
| RC + TRE |  | 0.03 | (-4.08, 4.14) | 0.9878 |
| RC + Ex |  | -3.83 | (-14.43, 6.77) | 0.4785 |
| HC + TRE | QUICKI | 0.005 | (-0.02, 0.03) | 0.7026 |
| HC + Ex |  | 0.014 | (-0.001, 0.03) | 0.0609 |
| RC + TRE |  | 0.017 | (0.003, 0.03) | 0.0157 |
| RC + Ex |  | -0.003 | (-0.03, 0.03) | 0.8395 |

^1^ A1c, hemoglobin A1c; CI, confidence interval; Ex, exercise; HC, high carbohydrate; RC, reduced carbohydrate; TRE, time restricted eating; QUICKI, quantitative insulin-sensitivity check index.

^2^Weighted and replicated regression was used to rank order the change in outcomes from baseline to week 16 follow-up across the four EAI treatment pathways.

**Supplemental Table 5.** Estimates for adaptive intervention groups for cardiometabolic outcomes (n=82).

| Embedded adaptive intervention^1^ | Outcome^1^ | Estimated Mean^2^ | CI^1^ | P |
| --- | --- | --- | --- | --- |
| HC + TRE | BF (kg) | -4.0 | (-6.61, -1.36) | 0.0030 |
| HC + Ex |  | -0.30 | (-1.75, 1.16) | 0.6894 |
| RC + TRE |  | -1.04 | (-2.51, 0.43) | 0.1658 |
| RC + Ex |  | -4.19 | (-7.1, -1.27) | 0.0049 |
| HC + TRE | SBP (mm Hg) | -8.62 | (-18.29, 1.05) | 0.0805 |
| HC + Ex |  | 5.49 | (-2.06, 13.03) | 0.1543 |
| RC + TRE |  | -0.33 | (-7.36, 6.7) | 0.9273 |
| RC + Ex |  | -16.54 | (-27.98, -5.11) | 0.0046 |
| HC + TRE | DBP (mm Hg) | -5.12 | (-10.22, -0.02) | 0.0492 |
| HC + Ex |  | 2.82 | (-1.9, 7.53) | 0.2413 |
| RC + TRE |  | -0.86 | (-6, 4.29) | 0.7444 |
| RC + Ex |  | -8.33 | (-14.64, -2.01) | 0.0098 |
| HC + TRE | TC (mg/dL) | -14.26 | (-25.86, -2.66) | 0.0160 |
| HC + Ex |  | -2.69 | (-12.95, 7.56) | 0.6067 |
| RC + TRE |  | -0.31 | (-9.46, 8.84) | 0.9475 |
| RC + Ex |  | -2.21 | (-16.97, 12.54) | 0.7688 |
| HC + TRE | HDL-C (mg/dL) | -4.48 | (-10.93, 1.97) | 0.1738 |
| HC + Ex |  | 2.45 | (-3.63, 8.54) | 0.4295 |
| RC + TRE |  | -4.33 | (-10.95, 2.3) | 0.2005 |
| RC + Ex |  | -5.21 | (-12.87, 2.46) | 0.1830 |
| HC + TRE | LDL-C (mg/dL) | -12.18 | (-26.73, 2.37) | 0.1010 |
| HC + Ex |  | -0.89 | (-10.1, 8.31) | 0.8490 |
| RC + TRE |  | 2.02 | (-6.11, 10.15) | 0.6266 |
| RC + Ex |  | -6.62 | (-22.83, 9.59) | 0.4234 |
| HC + TRE | TG (mg/dL) | 0.76 | (-39.6, 41.12) | 0.9706 |
| HC + Ex |  | -4.28 | (-24.52, 15.96) | 0.6785 |
| RC + TRE |  | -1.72 | (-24.47, 21.04) | 0.8824 |
| RC + Ex |  | -3.32 | (-50.42, 43.78) | 0.8901 |

^1^ BF, body fat; CI, confidence interval; DBP, diastolic blood pressure; Ex, exercise; HC, high carbohydrate; HDL-C, high-density lipoprotein cholesterol; LDL-C, low-density lipoprotein cholesterol; RC, reduced carbohydrate; SBP, systolic blood pressure; TC, total cholesterol; TG, triglycerides; TRE, time restricted eating.

^2^Weighted and replicated regression was used to rank order the change in outcomes from baseline to week 16 follow-up across the four EAI treatment pathways.

**Supplemental Table 6**. Sensitivity analysis^1^ results for weight loss (primary outcome).

| Group^2^ | Outcome | Estimate | 95% CI | P |
| --- | --- | --- | --- | --- |
| HC | Weight loss (kg) | -5.04 | (-6.73, -3.35) | <0.0001 |
| RC |  | -5.382 | (-7.22, -3.55) | <0.0001 |
| Between group differences (HC vs RC) |  | 0.342 | (-1.67, 2.35) | 0.7393 |
| Ex |  | -3.26 | (-5.31, -1.21) | 0.0018 |
| TRE |  | -3.632 | (-5.82, -1.44) | 0.0012 |
| Between group differences (Ex vs TRE) |  | 0.371 | (-1.54, 2.28) | 0.7037 |

^1^ In sensitivity analyses, models were adjusted for baseline values in addition to sex.

^2^ CI, confidence interval; Ex, exercise; HC, high carbohydrate; RC, reduced carbohydrate; TRE, time restricted eating.

**Supplemental Table 7**. Sensitivity analysis^1^ results for glycemic measures (secondary outcomes).

| Group^2^ | Outcome | Estimate | 95% CI | P |
| --- | --- | --- | --- | --- |
| HC | A1c (%) | -0.124 | (-0.227, -0.022) | 0.017 |
|  | Glucose (mg/dL) | -6.884 | (-11.02, -2.75) | 0.0011 |
|  | Insulin (U/mL) | -1.466 | (-3.58, 0.652) | 0.1749 |
|  | QUICKI | 0.007 | (-0.004, 0.017) | 0.202 |
| RC | A1c (%) | -0.176 | (-0.283, -0.068) | 0.0013 |
|  | Glucose (mg/dL) | -2.617 | (-6.91, 1.67) | 0.2317 |
|  | Insulin (U/mL) | -0.837 | (-3.04, 1.37) | 0.4573 |
|  | QUICKI | 0.007 | (-0.004, 0.017) | 0.2161 |
|  | Weight loss (kg) | -5.382 | (-7.22, -3.55) | <0.0001 |
| Between group differences (HC vs RC) | A1c (%) | 0.051 | (-0.070, 0.170) | 0.408 |
|  | Glucose (mg/dL) | -4.267 | (-8.83, 0.300) | 0.0669 |
|  | Insulin (U/mL) | -0.629 | (-2.95, 1.69) | 0.5952 |
|  | QUICKI | 1.51E-05 | (-0.011, 0.011) | 0.998 |
| Ex | A1c (%) | -0.143 | (-0.310, 0.023) | 0.0912 |
|  | Glucose (mg/dL) | -2.885 | (-10.08, 4.31) | 0.4318 |
|  | Insulin (U/mL) | -0.161 | (-3.61, 3.29) | 0.9269 |
|  | QUICKI | -0.001 | (-0.016, 0.014) | 0.8684 |
|  | Weight loss (kg) | -3.26 | (-5.31, -1.21) | 0.0018 |
| TRE | A1c (%) | -0.211 | (-0.394, -0.027) | 0.0245 |
|  | Glucose (mg/dL) | -8.708 | (-16.43, -0.988) | 0.0271 |
|  | Insulin (U/mL) | 0.846 | (-2.93, 4.62) | 0.6604 |
|  | QUICKI | 0.004 | (-0.012, 0.021) | 0.6106 |
| Between group differences (Ex vs TRE) | A1c (%) | 0.067 | (-0.103, 0.238) | 0.4379 |
|  | Glucose (mg/dL) | 5.824 | (-1.10, 12.75) | 0.0993 |
|  | Insulin (U/mL) | -1.007 | (-4.38, 2.37) | 0.5588 |
|  | QUICKI | -0.006 | (-0.021, 0.009) | 0.4628 |

^1^ In sensitivity analyses, models were adjusted for baseline values in addition to sex.

^2^ A1c, hemoglobin A1c; CI, confidence interval; Ex, exercise; HC, high carbohydrate; RC, reduced carbohydrate; QUICKI, quantitative insulin-sensitivity check index; TRE, time restricted eating.

**Supplemental Table 8**. Sensitivity analysis^1^ results for secondary outcomes.

| Group | Outcome | Estimate | 95% CI | P |
| --- | --- | --- | --- | --- |
| HC | BF (kg) | -1.917 | (-3.11, -0.720) | 0.0017 |
|  | SBP (mm Hg) | -1.748 | (-6.57, 3.08) | 0.4776 |
|  | DBP (mm Hg) | -0.745 | (-3.30, 1.81) | 0.5676 |
|  | TC (mg/dL) | -8.857 | (-16.72, -0.994) | 0.0273 |
|  | HDL-C (mg/dL) | -2.105 | (-6.53, 2.32) | 0.3515 |
|  | LDL-C (mg/dL) | -6.648 | (-13.14, -0.152) | 0.0449 |
|  | TG (mg/dL) | 0.348 | (-13.15, 13.84) | 0.9597 |
| RC | BF (kg) | -2.423 | (-3.71, -1.13) | <0.0001 |
|  | SBP (mm Hg) | -7.316 | (-12.68, -1.95) | 0.0075 |
|  | DBP (mm Hg) | -3.159 | (-6.00, -0.319) | 0.0293 |
|  | TC (mg/dL) | -1.61 | (-10.06, 6.84) | 0.7087 |
|  | HDL-C (mg/dL) | -5.047 | (-10.09, -0.001) | 0.05 |
|  | LDL-C (mg/dL) | -2.29 | (-9.28, 4.70) | 0.5208 |
|  | TG (mg/dL) | -0.008 | (-14.51, 14.50) | 0.9992 |
| Between group differences (HC vs RC) | BF (kg) | 0.506 | (-0.772, 1.78) | 0.4381 |
|  | SBP (mm Hg) | 5.568 | (-0.295, 11.43) | 0.0627 |
|  | DBP (mm Hg) | 2.414 | (-0.675, 5.50) | 0.1256 |
|  | TC (mg/dL) | -7.247 | (-16.32, 1.83) | 0.1175 |
|  | HDL-C (mg/dL) | 2.942 | (-2.40, 8.28) | 0.2804 |
|  | LDL-C (mg/dL) | -4.358 | (-11.88, 3.16) | 0.2559 |
|  | TG (mg/dL) | 0.356 | (-15.15, 15.86) | 0.9641 |
| Ex | BF (kg) | -1.483 | (-3.04, 0.071) | 0.0614 |
|  | SBP (mm Hg) | -2.976 | (-12.01, 6.06) | 0.5186 |
|  | DBP (mm Hg) | -1.902 | (-6.77, 2.97) | 0.4441 |
|  | TC (mg/dL) | 2.363 | (-8.84, 13.56) | 0.6791 |
|  | HDL-C (mg/dL) | -1.651 | (-10.09, 6.79) | 0.7014 |
|  | LDL-C (mg/dL) | 0.516 | (-9.66, 10.69) | 0.9208 |
|  | TG (mg/dL) | -14.363 | (-33.37, 4.64) | 0.1385 |
| TRE | BF (kg) | -1.901 | (-3.53, -0.270) | 0.0223 |
|  | SBP (mm Hg) | -5.15 | (-14.70, 4.40) | 0.2906 |
|  | DBP (mm Hg) | -2.206 | (-7.42, 3.00) | 0.4065 |
|  | TC (mg/dL) | -5.386 | (-17.45, 6.68) | 0.3815 |
|  | HDL-C (mg/dL) | -6.008 | (-15.00, 2.99) | 0.1905 |
|  | LDL-C (mg/dL) | -2.66 | (-13.78, 8.46) | 0.6392 |
|  | TG (mg/dL) | -7.521 | (-28.22, 13.18) | 0.4764 |
| Between group differences (Ex vs TRE) | BF (kg) | 0.418 | (-0.950, 1.79) | 0.5494 |
|  | SBP (mm Hg) | 2.175 | (-6.63, 10.98) | 0.6283 |
|  | DBP (mm Hg) | 0.304 | (-4.40, 5.01) | 0.8991 |
|  | TC (mg/dL) | 7.749 | (-2.84, 18.34) | 0.1516 |
|  | HDL-C (mg/dL) | 4.357 | (-3.94, 12.65) | 0.3032 |
|  | LDL-C (mg/dL) | 3.176 | (-6.64, 12.99) | 0.5258 |
|  | TG (mg/dL) | -6.842 | (-25.00, 11.31) | 0.4601 |

^1^ In sensitivity analyses, models were adjusted for baseline values in addition to sex.

^2^ BF, body fat; CI, confidence interval; DBP, diastolic blood pressure; Ex, exercise; HC, high carbohydrate; HDL-C, high-density lipoprotein cholesterol; LDL-C, low-density lipoprotein cholesterol; RC, reduced carbohydrate; SBP, systolic blood pressure; TC, total cholesterol; TG, triglycerides; TRE, time restricted eating.

**Supplemental Figure 1.** Box plots for the change in weight for each weight loss class.


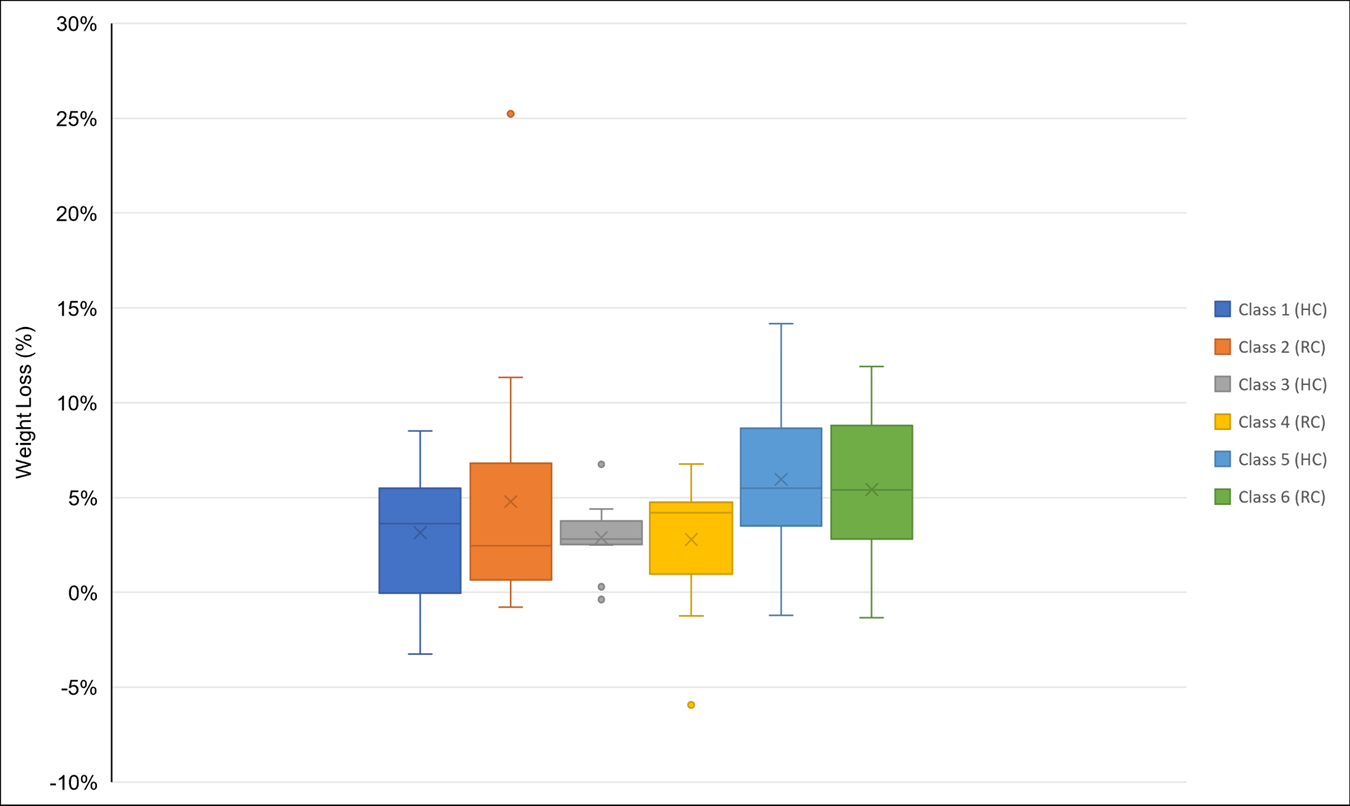

Supplement: Appendix A. [file NIHMS2157602-supplement-Appendix_A_.docx]
